# Consumption of out-of-season orange modulates fat accumulation, morphology and gene expression in the adipose tissue of Fischer 344 rats

**DOI:** 10.1007/s00394-019-01930-9

**Published:** 2019-02-20

**Authors:** Albert Gibert-Ramos, Hector Palacios-Jordan, M. Josepa Salvadó, Anna Crescenti

**Affiliations:** 1 Nutrigenomics Research Group, Department of Biochemistry and Biotechnology, Universitat Rovira i Virgili, Campus Sescelades, Building N4, Marcel·lí Domingo 1, 43007 Tarragona, Spain; 2 Eurecat, Centre Tecnològic de Catalunya, Unitat de Nutrició i Salut, Reus, Spain

**Keywords:** Obesity, Xenohormesis, White adipose tissue, Brown adipose tissue, Photoperiod, Seasonality

## Abstract

**Purpose:**

According to the xenohormesis theory, animals receive signals from plants that give clues about the changing environment, and thus, depending on the season of the year, animals develop physiological changes to adapt in advance to the seasonal changes. Our objective was to study how the same fruit cultivated during two different seasons could affect the adipose tissue of rats.

**Methods:**

Thirty-six Fischer 344 rats were acclimated for 4 weeks to long-day or short-day (SD) photoperiods. After adaptation, three groups (*n* = 6) from each photoperiod were supplemented either with orange from the northern (ON) or southern (OS) hemispheres harvested in the same month or a vehicle (VH) for 10 weeks. Biometric measurements, postprandial plasmatic parameters, gene expression of the inguinal white adipose tissue (IWAT) and brown adipose tissue (BAT), and the histology of the IWAT were analysed.

**Results:**

The OSSD group increased its fat content compared to the VHSD, while the ON groups showed no biometric differences. The OS groups were further studied, and the IWAT showed increased levels of *Pparγ* gene expression and a higher percentage of larger adipocytes compared to the VH group. The BAT showed down-regulation of *Lpl, Cpt1b* and *Pparα* in the OSSD group compared to that in the VHSD group, suggesting an inhibition of BAT activity, however, *Ucp1* gene expression was up-regulated.

**Conclusions:**

We observed a different effect from both fruits, with the OS promoting a phenotype prone to fat accumulation when consumed in an SD photoperiod, which might be explained by the xenohormesis theory.

**Electronic supplementary material:**

The online version of this article (10.1007/s00394-019-01930-9) contains supplementary material, which is available to authorized users.

## Introduction

The xenohormesis theory posits that plants synthesise small molecules or secondary metabolites when under a mild stress and that these molecules are detected by heterotrophs when ingested, activating a response that allows them to adapt to this new environment to survive [1, 2]. For example, this can be activating energy accumulation when food is scarce or triggering reproductive changes during good weather [3, 4]. This molecular signature can provide information about the season of the year and can be defined by different factors that modify the secondary metabolite content, including the polyphenol content of fruits, such as temperature, sunlight, and access to water [5–8].

Evolution has made animals sensitive to seasons, i.e., predictable events that change the environment in a way that affects their survival. Pre-adaptation to the coming season is achieved via the molecular clock. This mechanism generates circadian rhythms and synchronizes the external light–dark cycle with many processes using an autoregulatory feedback loop that has a periodicity of 24 h and is essential for the sleep–wake cycle of animals [9]. The central clock is the master regulator of this process, and it is found in the suprachiasmatic nucleus (SCN) of the hypothalamus and is principally entrained by ambient light [10]. Moreover, the SCN signals peripheral tissues with hormones, the autonomic nervous system and behavioural pathways, which entrain the peripheral clocks [11]. However, it has been reported that these peripheral clocks can also be synchronized independently from the central clock by food intake [12]. The disruption of the molecular clock can result in several health problems such as obesity or metabolic syndrome [13]. For example, artificial lightning has been proposed as a disruptor of circadian synchrony through misaligning the photoperiod cycle [14]. Additionally, people who usually work a night shift have been found to have a higher occurrence of metabolic syndrome [15, 16]. Furthermore, even though an excess of energy intake is marked as the principal contributor to an increase in fat accumulation in obesity, some authors disagree as to whether this is the main driver in obesity and whether other environmental factors such as climate, artificial light and day length could be involved [17, 18].

The increasing international exchange of goods allows populations to buy food from distant countries. This way, we are able to eat seasonal fruits all year long, independent of the season of the consumer or at a lower economical cost. For example, in 2015, 31.3% of citrus fruits that entered the European Union market were from South Africa and an additional 11.2% came from Argentina [19], which are both southern hemisphere countries.

Considering all of the evidence above together with the xenohormesis theory raises the question of whether the consumption of fruit coming from a different season from the current one of the consumer could somehow have a metabolic effect on an organism by giving erroneous signals of the photoperiod. The hypothesis of this study was that two sweet oranges of the navelina variety from both hemispheres and harvested in the same month, would have different effects on the rat adipose tissue due to differences in the farming season and the photoperiod of consumption. The objective was to investigate how two oranges harvested during two different seasons can affect adipose tissue physiology and metabolism of Fischer 344 rats depending on the photoperiod of consumption.

## Methods

### Animals and treatments

The animals used were 36 2-month-old male Fischer 344/IcoCrl rats (Charles River Laboratories, Barcelona, Spain) fed with a standard chow diet (Panlab, Barcelona, Spain). The animals were housed singly at 22 °C and 55% humidity with free access to food and water. The animals were randomly distributed into six groups (*n* = 6) depending on the treatment received and the photoperiod to which they were exposed. Distribution was balanced measuring body weight, so at the start of the experiment, the mean and SEM of this parameter were statistically the same among groups. All animal studies have been approved by the Animal Ethics Committee of the University Rovira i Virgili (Tarragona, Spain) (reference number 4249) and have therefore been performed in accordance with the ethical standards laid down in the 1964 Declaration of Helsinki and its later amendments.

Animals were acclimatised to two photoperiods with different light:dark cycles, long day (LD; 18 h light: 6 h dark) and short day (SD; 6 h light: 18 h dark) for 4 weeks. After the adaptation period to each of the photoperiods (SD and LD), three groups of animals were treated daily with 100 mg/kg body weight lyophilized sweet orange (O) (*Citrus x sinsensis*) from the northern hemisphere (ON) or from the southern hemisphere (OS) or with 20 mg/kg body weight of the vehicle (VH) for 10 weeks. The VH treatment (1:1, glucose: fructose solution) was used to match the sugar consumption of those receiving the orange treatments. Accordingly, the six animal groups of the study were: ONSD (Orange North Hemisphere Short Day), OSSD (Orange South Hemisphere Short Day), VHSD (Vehicle Short Day), ONLD (Orange North Hemisphere Long Day), OSLD (Orange South Hemisphere Long Day), and VHLD (Vehicle Long Day). Both oranges were of the navelina variety and were harvested during the same month. ON was cultivated in Spain and OS was cultivated in Argentina. Both fruits were bought from a local hypermarket, frozen in liquid nitrogen, ground with a blender and freeze dried with a lyophiliser. Afterwards, the powder was stored at room temperature and protected from light until use. The characterization of both sweet oranges used in this experiment was performed using an HPLC–ESI–MS/MS methodology, and is described by Iglesias-Carres et al. [20]. The most prominent phenolic compound of both oranges was hesperidin (38.28 × 10^3^ mg/kg dry weight (dw) for ON and 25.83 × 10^3^ mg/kg dw for OS), followed by narirutin (8.07 × 10^3^ mg/kg dw for ON and 3.54 × 10^3^ mg/kg dw for OS). Other compounds such as didymin, kaempferol-3-O-rutinoside, protocatechuic acid *O*-glucoside, among others, were also found in relevant concentrations and in accordance to the characterization of other sweet orange varieties [20].

The body weight and food intake for each animal were recorded every week. One week prior to sacrifice, the fat mass and lean mass were analysed by quantitative magnetic resonance using an EchoMRI-700™ (Echo Medical Systems, LLC., TX, USA) without anaesthesia. After 10 weeks of treatment, animals were sacrificed in the fed state by decapitation, and blood was collected from the neck, stored at room temperature for 45 min and then centrifuged at 1200*g* for 10 min to collect the serum. Different white adipose tissue (WAT) depots—epididymal (EWAT), retroperitoneal (RWAT), inguinal (IWAT) and mesenteric (MWAT)—as well as interscapular brown adipose tissue (BAT) were rapidly removed after death, weighed, frozen in liquid nitrogen and stored at − 80° until further analysis. Adiposity was determined by an adiposity index computed for each rat as the sum of EWAT, IWAT, MWAT and RWAT deposit weight and expressed as a percentage of total body weight.

The Animal Ethics Committee of the University Rovira i Virgili (Tarragona, Spain) approved all of the procedures, and the guidelines for the use and care of laboratory animals of the university were followed.

### Plasma analysis

Enzymatic colorimetric kits were used for the determination of plasma glucose, triglyceride, cholesterol (QCA, Barcelona, Spain) and non-esterified free fatty acid (NEFA) (WAKO, Neuss, Germany) levels. Insulin and leptin levels were quantified with a rat-specific enzyme immunoassay kit (Millipore, Madrid, Spain).

### RNA extraction and quantification by real-time qRT–PCR

Total RNA from IWAT and BAT tissues was extracted using Trizol^®^ reagent (Thermo Fisher, Madrid, Spain) following the manufacturer’s instructions. RNA yield was quantified using a Nanodrop ND-1000 spectrophotometer (NanoDrop Technologies, Willmington, DE, USA), and the integrity of the RNA was confirmed using agarose gel electrophoresis.

Then, 0.5 µg of total RNA was reverse transcribed using the high-capacity cDNA Reverse Transcription Kit (Applied Biosystems, Madrid, Spain) in a Multigene Thermal Cycler (Labnet, Madrid, Spain), and for Q-PCR, the CFX96 real-time system C1000 Touch Thermal Cycler (Bio-Rad, Barcelona, Spain) with the iTaq™ Universal SYBR^®^ Green Supermix (Bio-Rad, Barcelona, Spain) was used. All Q-PCRs were performed with the following cycling conditions after an initial Taq activation at 95 °C for 30 s: 39 cycles of 95 °C for 5 s and 60 °C for 30 s. A melt curve was produced after the previous steps by increasing the temperature from 65 to 95 °C by 0.5 °C every 5 s. Gene expression levels in IWAT tissue were analysed for the acetyl-CoA carboxylase alpha (*Acacα*), fatty acid synthase (*Fasn*), glycerol-3-phosphate acyltransferase (*Gpat*), monoglyceride lipase (*Mgll*), adipose triglyceride lipase (A*tgl*), hormone-sensitive lipase (*Hsl*), CCAAT/enhancer-binding protein alpha (*CEBP*α) and peroxisome proliferator-activated receptor gamma (*Pparγ*) genes. In BAT tissue, we measured the gene expression levels for the cluster of differentiation 36 (*Cd36*), fatty acid transport protein 1 (*Fatp1*), lipoprotein lipase (*Lpl*), carnitine palmitoyltransferase 1B (*CPT1b*), hydroxyacil-CoA dehydrogenase (*Had*) and peroxisome proliferator-activated receptor alpha (*Pparα*) genes. Furthermore, we measured the gene expression levels for PR domain-containing 16 (*Prdm16*) and uncoupling protein 1 (*Ucp1*) in both tissues. The primers for the different genes are described in Supplementary Table 1 (Online resource 1) and were obtained from Biomers.net (Ulm, Germany). The relative expression of each mRNA was calculated as a percentage of the vehicle group using the 2^−∆∆Ct^ method [21] with *Ppia, Actb* and *Hprt1* as reference genes. Each PCR was performed at least in duplicate.

### Western blot

The Ucp1 protein content in BAT was determined by Western blot. Tissues were homogenized in RIPA (radio immunoprecipitation assay lysis buffer), and the protein was extracted and stored at − 20 °C. The protein content was quantified using a BCA protein assay kit (Pierce, Rockford, IL, USA) following manufacturer’s instructions.

First, 15 µg of protein in Laemmli loading buffer was denatured, loaded onto 10% acrylamide gels made with TGX™ Fast Cast™ Acrylamide Solutions (Bio-Rad, Barcelona, Spain) and run at 90 V for 75 min. Gels were then transferred onto a PVDF membrane using the Trans-Blot Transfer System (Bio-Rad, Barcelona, Spain) with Trans-Blot Turbo Mini PVDF Transfer Packs (Bio-Rad, Barcelona, Spain) following the manufacturer’s instructions. The membrane was blocked and then incubated with anti-Ucp1 antibody (Abcam, Cambridge, United Kingdom) at 4 °C overnight. Afterwards, the membrane was incubated for 2 h with the secondary antibody (GE Health Care Life Sciences, Barcelona), and the protein was detected with the chemiluminescent reagent ECL Select Western Blotting Detection Reagent (GE Healthcare, Barcelona, Spain). Protein levels were quantified with the open source software ImageJ [22] and normalized to β-actin protein levels.

### Histology

For histological analyses, frozen IWAT samples were thawed and fixed in 4% formaldehyde. The tissue underwent successive dehydration and paraffin infiltration immersion (Citadel 2000, HistoStar, Thermo Scientific, Madrid, Spain), and the paraffin blocks were cut into 2-µm-thick sections using a microtome (Microm HM 355S, Thermo Scientific). The sections were then subjected to automated haematoxylin–eosin staining (Varistain Gemini, Shandom, Thermo Scientific) [23].

Sections were observed and acquired at x10 magnification using AxioVision Zeiss Imaging software (Carl Zeiss Iberia, S.L., Madrid, Spain). The area and number of adipocytes were measured using the open source software Adiposoft (CIMA, University of Navarra, Spain). Four fields per sample and six samples from each group were measured. The area was calculated from the average value of the area in all measured fields for each group. The total adipocyte number was calculated using the formula: $\left( {\frac{\pi }{6}} \right) \times \left( {3{\sigma ^2} \times \bar {d}+{{\bar {d}}^3}} \right),$ where $\stackrel{-}{d}$ is the mean diameter and σ is the standard deviation of the diameter, to obtain an average adipocyte volume [24]. Afterwards, we converted this value to the average adipocyte weight using the adipocyte density (0.92 g/ml), and to obtain the total adipocyte number, and the weight of the IWAT deposit was divided by the average adipocyte weight, as proposed by *Lemonnier* [25]. Frequencies of adipocytes were obtained by distributing cells into two groups according to their area (< 5000 µm^2^ or > 5000 µm^2^) and calculating the percentage relative to the total number of counted cells.

### Statistical analysis

The software IBM SPSS (SPSS Inc, Chicago, IL, USA) was used for statistical analysis. Data are expressed as the mean ± SEM, and significant differences were analysed by one-way ANOVA, followed by Duncan’s multiple range test with post hoc comparison between all groups, comparing separately both oranges with the vehicle. mRNA expression levels in BAT were analysed using Student’s *t* test. A *p* value ≤ 0.05 was considered statistically significant.

## Results

### Biometric and plasma parameters

In reference to the consumption of orange from the southern hemisphere, the OSSD group showed a significantly increased fat (gr, %) content compared to that of the VHSD group. We observed a similar effect on the adiposity index, i.e., the OSSD group showed a higher adiposity index than the VHSD group, although the differences between the groups were not significant (*p* = 0.10). No other differences were observed among the other parameters (Table 1). Furthermore, the consumption of orange from the northern hemisphere showed no significant differences for any parameters between the orange and vehicle-treated groups in any of the photoperiods (Table 2). Neither of the parameters in serum showed significant differences between the orange and vehicle-treated groups for any of the oranges studied in any of the photoperiods. No significant differences were found in the body weight and food intake between the orange and vehicle-treated groups for any of the oranges studied in any of the photoperiods (Tables 1, 2).

**Table 1:** Biometric and plasmatic measures of rats supplemented with orange lyophilizate from the southern hemisphere or vehicle in long-day and short-day photoperiods

	VHLD	OSLD	VHSD	OSSD
Weight (g)	386.5 ± 12.66	383.6 ± 4.41	370.33 ± 10.99	378.33 ± 10.9
Accumulated caloric intake (Kcal)	504.79 ± 11.67	484.27 ± 8.35	507.07 ± 0.43	496.93 ± 11.98
Fat (g)	55.64 ± 4.41^a^	54.78 ± 2.06^a^	45.06 ± 1.29^b^	53.56 ± 2.28^a^
Lean (g)	309.74 ± 8.96	298.7 ± 9.1	295.76 ± 8.19	299.24 ± 9.91
Fat (%)	14.38 ± 0.75^a^	14.79 ± 0.56^a^	12.52 ± 0.34^b^	14.18 ± 0.41^a^
Lean (%)	80.71 ± 0.67	80.44 ± 0.5	80.94 ± 1.04	79.22 ± 0.32
BAT (g)	0.35 ± 0.04	0.36 ± 0.01	0.47 ± 0.16	0.4 ± 0.1
EWAT (g)	12.02 ± 0.94^a^	11.76 ± 0.4^ab^	9.65 ± 0.67^c^	9.8 ± 0.37^bc^
MWAT (g)	8.04 ± 0.85	7.85 ± 0.44	6.53 ± 0.82	6.75 ± 0.26
IWAT (g)	5.82 ± 0.73	4.92 ± 0.57	4.52 ± 0.53	5.88 ± 0.77
RWAT (g)	10 ± 0.67^ab^	10.4 ± 0.45^a^	8.52 ± 0.58^b^	9.17 ± 0.48^ab^
Adiposity index (%)	9.22 ± 0.49^a^	9.42 ± 0.17^a^	7.72 ± 0.47^b^	8.37 ± 0.38^ab^
BAT (%)	0.092 ± 0.01	0.097 ± 0.004	0.086 ± 0.017	0.105 ± 0.023
EWAT (%)	3.09 ± 0.17^a^	3.14 ± 0.12^a^	2.6 ± 0.14^b^	2.59 ± 0.07^b^
MWAT (%)	2.06 ± 0.17	2.12 ± 0.13	1.74 ± 0.16	1.78 ± 0.05
IWAT (%)	1.5 ± 0.15	1.37 ± 0.14	1.25 ± 0.14	1.57 ± 0.22
RWAT (%)	2.58 ± 0.11^ab^	2.79 ± 0.1^a^	2.41 ± 0.05^b^	2.43 ± 0.12^b^
Glucose (mmol/L)	8.65 ± 0.47	8.16 ± 0.12	7.83 ± 0.2	8.66 ± 0.16
Triglycerides (mg/dl)	142.06 ± 7.74	152.61 ± 9.49	197.9 ± 14.45	174.57 ± 8.56
Cholesterol (mmol/L)	4.73 ± 0.07	4.26 ± 0.19	4.06 ± 0.33	4.14 ± 0.17
NEFA (mg/dl)	22.49 ± 3.21	21.09 ± 0.59	23.34 ± 1.74	25.01 ± 1.42
Insulin (ng/ml)	5.54 ± 0.73	5.22 ± 0.68	4.04 ± 0.66	4.39 ± 0.63
Leptin (ng/ml)	18.56 ± 0.31	19.93 ± 0.98	16.59 ± 1.49	17.38 ± 1.22

Fischer 344 rats supplemented with orange lyophilizate from the southern hemisphere (OS) or vehicle (VH) in long-day (LD) and short-day (SD) photoperiods. The adiposity index was computed as the sum of EWAT, MWAT, IWAT and RWAT deposit weights and expressed as a percentage of total body weight. Data are presented as the mean ± SEM and the four groups were compared by one-way ANOVA (*p* < 0.05) followed by Duncan’s new multiple range (MRT) post hoc test

*BAT* Interscapular brown adipose tissue, *EWAT* epididymal white adipose tissue, *MWAT* mesenteric white adipose tissue, *IWAT* inguinal white adipose tissue, *RWAT* retroperitoneal white adipose tissue

**Table 2:** Biometric and plasmatic measures of rats supplemented with orange lyophilizate from the northern hemisphere or vehicle in long- and short-day photoperiods

	VHLD	ONLD	VHSD	ONSD
Weight (g)	386.5 ± 12.66	380.33 ± 11.33	370.33 ± 10.99	359,83 ± 8,68
Accumulated caloric intake (Kcal)	504.79 ± 11.67	480.64 ± 5.88	507.07 ± 0.43	492.15 ± 0,28
Fat (g)	55.64 ± 4.41	54.14 ± 3.67	45.06 ± 1.29	49,74 ± 4,01
Lean (g)	309.74 ± 8.96	305,24 ± 8,52	295.76 ± 8.19	284,49 ± 9,44
Fat (%)	14.38 ± 0.75	14,25 ± 0,8	12.52 ± 0.34	13,9 ± 1,19
Lean (%)	80.71 ± 0.67	80,54 ± 0,78	80.94 ± 1.04	79,26 ± 1
BAT (g)	0.35 ± 0.04	0,29 ± 0,038	0.47 ± 0.16	0,395 ± 0,13
EWAT (g)	12.02 ± 0.94	11,98 ± 0,89	9.65 ± 0.67	9,53 ± 0,93
MWAT (g)	8.04 ± 0.85	7.92 ± 0.62	6.53 ± 0.82	5.95 ± 0.62
IWAT (g)	5.82 ± 0.73	5.62 ± 0.63	4.52 ± 0.53	5.09 ± 0.6
RWAT (g)	10 ± 0.67^a^	9.9 ± 0.36^a^	8.52 ± 0.58^b^	8.93 ± 0.6^ab^
Adiposity index (%)	9.22 ± 0.49	9.29 ± 0.42	7.72 ± 0.47	8.21 ± 0.71
BAT (%)	0.092 ± 0.01	0.077 ± 0.011	0.086 ± 0.017	0.111 ± 0.036
EWAT (%)	3.09 ± 0.17	3.13 ± 0.16	2.6 ± 0.14	2.65 ± 0.26
MWAT (%)	2.06 ± 0.17	2,08 ± 0,15	1.74 ± 0.16	1.66 ± 0.19
IWAT (%)	1.5 ± 0.15	1.48 ± 0.16	1.25 ± 0.14	1.41 ± 0.16
RWAT (%)	2.58 ± 0.11	2.6 ± 0.05	2.41 ± 0.05	2.49 ± 0.18
Glucose (mmol/L)	8.65 ± 0.47	8.21 ± 0.15	7.83 ± 0.2	8.18 ± 0.39
Triglycerides (mg/dl)	142.06 ± 7.74	135.62 ± 8.41	197.9 ± 14.45	165.92 ± 19.86
Cholesterol (mmol/L)	4.73 ± 0.07	4.27 ± 0.09	4.06 ± 0.33	4.16 ± 0.25
NEFA (mg/dl)	22.49 ± 3.21	20.86 ± 2.91	23.34 ± 1.74	25.74 ± 2.25
Insulin (ng/ml)	5.54 ± 0.73	5.65 ± 0.08	4.04 ± 0.66	4.16 ± 0.32
Leptin (ng/ml)	18.56 ± 0.31	18.79 ± 1.52	16.59 ± 1.49	18 ± 2.75

Fischer 344 rats supplemented with orange lyophilizate from the northern hemisphere (ON) or vehicle (VH) in long-day (LD) and short-day (SD) photoperiods. The adiposity index was computed as the sum of EWAT, MWAT, IWAT and RWAT deposit weights and expressed as a percentage of total body weight. Data are presented as the mean ± SEM and the four groups were compared by one-way ANOVA (*p* < 0.05) followed by Duncan’s new multiple range (MRT) post hoc test

*BAT* Interscapular brown adipose tissue, *EWAT* epididymal white adipose tissue, *MWAT* mesenteric white adipose tissue, *IWAT* inguinal white adipose tissue, *RWAT* retroperitoneal white adipose tissue

### Gene expression in IWAT tissue

We decided to analyse the gene expression levels of genes related to adiposity and thermogenesis in the IWAT deposits of the VH and OS groups despite the few changes observed in their biometric parameters. In this sense, it should be taken into account that we observed a clear effect from OS consumption on fat weight in the SD photoperiod. Furthermore, other studies have demonstrated that, even if no differences in weight between groups are found, differences in other parameters in the tissue can occur [26, 27]. Additionally, it has been described that IWAT deposits in adipose tissue have higher levels of browning compared to those of other WAT deposits [28, 29].

*PPARγ* mRNA expression in OSSD rats was significantly higher than that of the VHSD rats. No other differences were observed in any other gene (Table 3).

**Table 3:** mRNA expression levels in IWAT of rats supplemented with orange lyophilizate from the southern hemisphere (OS) or vehicle on long- and short-day photoperiods

	VHLD	OSLD	VHSD	OSSD
*Acacα*	122.3 6 ± 16.76	128.23 ± 2.73	100 ± 27.29	95.81 ± 7.78
*Fasn*	111.18 ± 30.69	122.82 ± 54.01	100 ± 13.85	107.21 ± 39.12
*Gpat*	133.31 ± 16.34	172.39 ± 20.4	100 ± 31.87	133.38 ± 47.1
*Mgll*	150.95 ± 25.2	191.08 ± 36.21	100 ± 37.81	158.04 ± 74.66
*Atgl*	172.23 ± 26.87^ab^	207.27 ± 32.01^a^	100 ± 35.57^b^	109.88 ± 38.05^ab^
*Hsl*	136.89 ± 18.55	167.51 ± 27.71	100 ± 28.92	108.53 ± 38.08
*c*/*Ebpα*	121.23 ± 16.4	167.75 ± 27.44	100 ± 26.55	99.93 ± 37.61
*Pparγ*	123.91 ± 3.45^ab^	128.55 ± 7.39^a^	100 ± 11.46^b^	129.23 ± 11.07^a^
*Prdm16*	111.92 ± 21.46	118.25 ± 10.27	100 ± 16.65	91.53 ± 25.77
*Ucp1*	102.2 ± 16.47	192.46 ± 63.84	100 ± 29.72	121.71 ± 21.79

Expression of genes related with lipogenesis, lipolysis, adipogenesis and thermogenesis in Fischer 344 rats supplemented with orange lyophilizate from the southern hemisphere (OS) or vehicle (VH) held in a long-day (LD) and short-day (SD) photoperiods. Data is presented as the ratios of gene expression, relative to β-actin, ppia and hprt and expressed as a percentage of the VHSD group, set at 100%. Results are presented as the mean ± SEM and data compared by one-way ANOVA (*p* < 0.05) followed by Duncan’s new multiple range (MRT) post hoc test

### Histology of IWAT

**Fig. 1:**
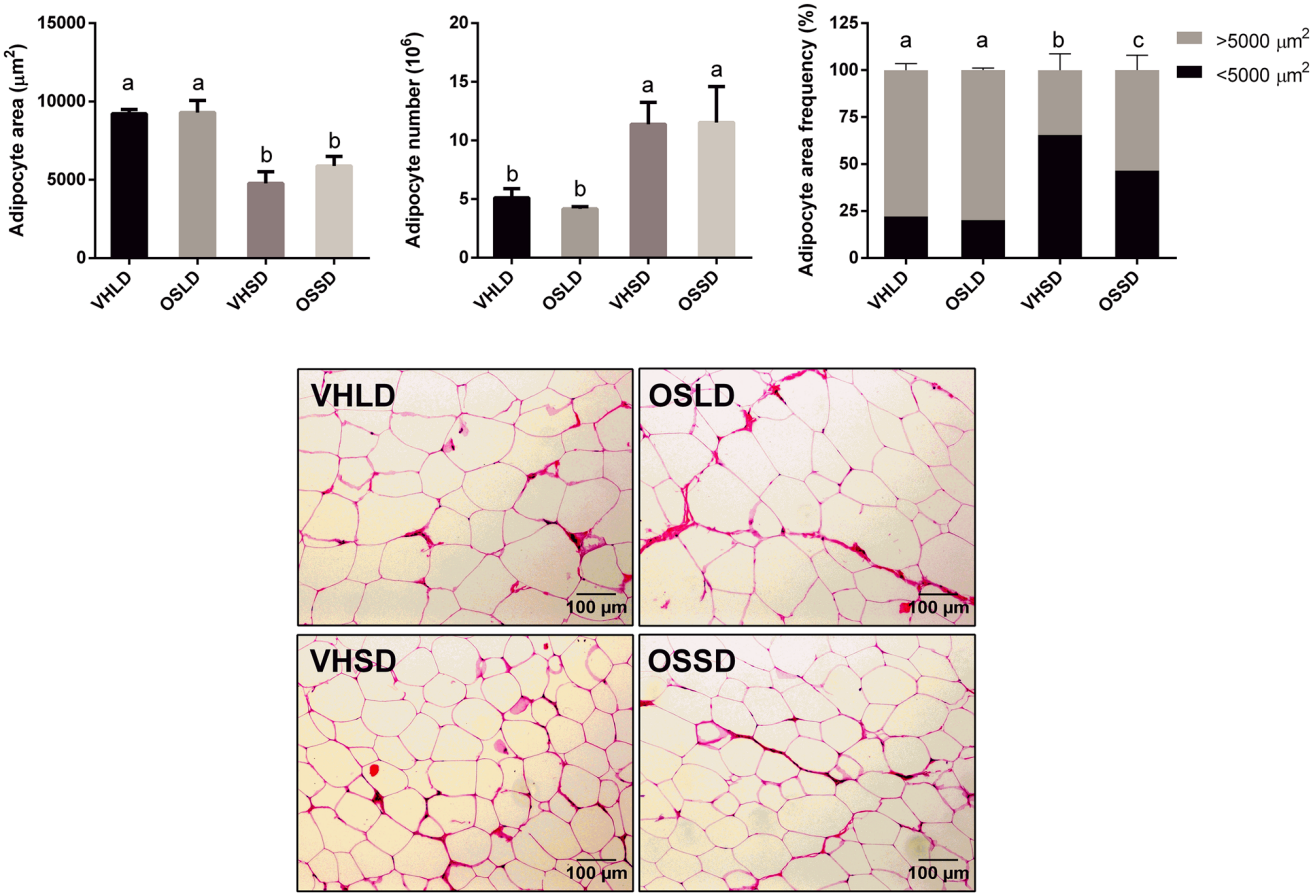
Cell number, cell area and adipocyte frequencies of IWAT of Fischer 344 rats supplemented with orange lyophilizate from the southern hemisphere (OS) in short-day (SD) or long-day (LD) photoperiods. For frequencies, adipocytes were distributed in two groups according to their area (< 5000 or > 5000 µm^2^). Data are presented as the mean ± SEM and statistical significance is analysed by one-way ANOVA (*p* < 0.05) followed by Duncan’s new multiple range (MRT) post hoc test

The OSSD group showed significantly higher levels of larger adipocytes (> 5000 µm^2^) than the VHSD rats, while no differences in adipocyte frequencies were observed between the LD groups (Fig. 1).

Adipocyte area and total number were not significantly different among groups.

### Gene expression in BAT tissue

Due to the effect of OS consumption on fat weight in the SD photoperiod, we analysed the expression of several genes related to BAT activity in this photoperiod. *Lpl* mRNA expression in OSSD rats was significantly down regulated compared to that in VHSD rats. Concerning β-oxidation, the gene expression levels of *Cpt1b* and *Pparα* were significantly down-regulated and those of *Ucp1* were highly up-regulated in the OSSD rats compared to those in the VHSD rats (Table 4).

**Table 4:** mRNA expression levels in BAT of rats supplemented with orange lyophilizate from the southern hemisphere or vehicle in a short-day photoperiod

	VHSD	OSSD
*Cd36*	100 ± 10	88.72 ± 10.87
*Fatp1*	100 ± 19.16	79.45 ± 8.76
*Lpl*	100 ± 6.68	59.21 ± 13.69*
*Cpt1b*	100 ± 10.81	57.47 ± 10.03*
*Had*	100 ± 5.58	85.89 ± 13.97
*Pparα*	100 ± 10.79	54.81 ± 6.73*
*Ucp1*	100 ± 5.98	210.43 ± 35.59*
*Prdm16*	100 ± 13.77	104.74 ± 20.49

Expression of genes in BAT related with β-oxidation, lipid uptake and thermogenesis in Fischer 344 rats supplemented with orange lyophilizate from the southern hemisphere (OS) or vehicle (VH) held in a short-day (SD) photoperiod. Data are presented as the ratios of gene expression relative to *Actb, Ppia* and *Hprt*, and expressed as a percentage of the VHSD group, set at 100%. Results are presented as the mean ± SEM and data compared by Student’s *t* test (p < 0.05)

### UCP1 protein levels on BAT

**Fig. 2:**
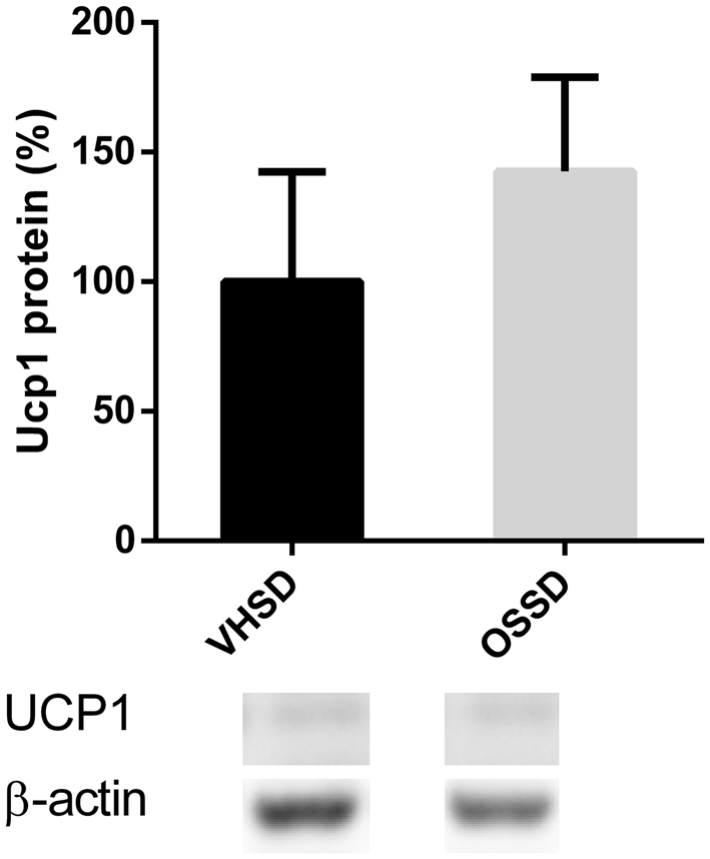
UCP1 protein levels in BAT measured by Western blotting of Fischer 344 rats supplemented with orange lyophilizate from the southern hemisphere (OS) or vehicle (VH) held in a short-day photoperiod (SD). Data are normalized to β-actin and to the VHSD group, set at 100%. Results are presented as the mean ± SEM and data compared with Student’s *t* test (*p* < 0.05)

No significant differences between the OSSD and VHSD groups were observed in the BAT UCP1 protein levels (Fig. 2).

## Discussion

In the globalized world, it is increasingly common to purchase consumables from distant countries. This practice has extended to food and, in particular, to fruits and vegetables due to more economical costs or preferences. This phenomenon has permitted the population to consume fruits during what is called “out of season”, which means consuming a fruit from a season that does not correspond to the current season of the consumer. According to the xenohormesis theory [1, 2], heterotrophs can recognize phenolic compounds and other chemical cues from autotrophs, which modify their molecular signatures depending on the environment, signalling animals about different stresses and conditions in the habitat, which can change their strategy for survival to adapt to the changing environment. This means that the consumption of fruit from a specific season or photoperiod could have different effects on the metabolism of different tissues, for example adipose tissue, depending on the season of consumption. Based on this theory, we performed this study to investigate how the same species of orange grown during two different seasons can affect adipose tissue physiology and metabolism depending on the photoperiod of consumption.

In the present study, the consumption of orange from the southern hemisphere by SD rats increased the body fat mass of rats compared to that of the control group in the same photoperiod, while it had no effect on the LD animals. These results seem to indicate that the consumption of orange harvested in the spring has the capacity to alter fat mass when consumed during a short photoperiod. These changes were dependent on the photoperiod, because only the animals that ate orange in the short day had their parameters altered, but it was also dependent on the season in which the fruit was harvested, because no differences were obtained with the orange from the northern hemisphere. Numerous studies in small rodents have shown that a long photoperiod increases fat accumulation or body weight, while the opposite was observed for a short photoperiod [30–34]. Our data show a similar effect on OSSD animals, which are in a short photoperiod but consuming fruit harvested in the spring. These results are in accordance with our hypothesis, suggesting that rats consuming orange out of season in a short photoperiod receive signals that a long photoperiod is approaching, meaning that they will adapt to the coming environment and adopt an advantageous phenotype, which translates into increased energy storage. According to *Lisard et al*., the type of phenolic compounds detected in each orange was practically the same, while the total amount of each was different. Mainly, ON had more quantity of each detected polyphenol than OS, expected to be caused by the different environmental conditions in which both oranges were harvested [20]. Thus, we believe that these differences in composition could be the ones responsible, together with the photoperiod, of the observed changes.

The reason why rodents increase their body mass in an LD photoperiod is not fully understood. Some authors report an increase in calorie intake or a change in diet preferences with longer photoperiods [31, 32], while other studies attribute the changes to a decrease in BAT activity [35] or to activation of WAT lipolysis and browning during the SD [33]. In our study, the possibility of an increase in the fat content of the OSSD group due to diet was discarded, because no significant changes were observed in food intake or plasmatic parameters. For this reason, the gene expression profiles of the IWAT and BAT were studied. We chose IWAT because it has been reported to present higher levels of browning than other typically studied white deposits [28, 29]. Moreover, even though no biometrical differences were detected, differences in gene expression or histology can still occur, as other authors have reported [26, 27]. Interestingly, in our study, the gene expression levels of PPARγ in IWAT tissue increased in the OSSD group compared to that in the control and VHSD groups, which would contribute to the differences detected in body fat mass. Even though no differences were detected in the other genes analysed, the PPARγ gene is known to activate the differentiation of preadipocytes into mature adipocytes, adipocyte lipid metabolism and the recruitment of bone marrow-derived circulating progenitor cells to WAT [36–38]. Therefore, this gene has a potential role in the activation of metabolic pathways related to lipid and fat accumulation in adipose tissue. The increased lipogenic capacity of the IWAT in the OSSD group was supported by the histology outcomes, since the OSSD animals had a higher number of larger adipocytes than the control group. These results are important and relevant, since other authors have suggested that there is a relationship between adipocyte size and the secretion of proinflammatory adipokines in subcutaneous fat deposits in healthy humans [39, 40], increasing the expression of proinflammatory adipokines with the size of the adipocytes. In fact, it is agreed that the size and number of white adipocytes are related to a metabolically healthy/unhealthy pattern. Thus, an increase in adipocyte size is related to insulin resistance and type II diabetes [41–43], while smaller adipocytes in obese patients are correlated with a healthier metabolic profile [44]. Furthermore, it is well known that chronic inflammation of adipose tissue is well correlated with metabolic syndrome and obesity [45, 46]. Thus, although the rats in our study did not show any signs of obesity, the possibility of a higher risk of obesity should be further studied. Overall, our results indicate that the consumption of oranges harvested in the spring in an SD increases fat accumulation and adipogenesis compared to the control group in the same photoperiod, and the different effects on the tissue depend on the photoperiod.

As explained before, since OS is a fruit with a molecular signature of an LD, it could stimulate effects in the BAT of SD rats, similar to those observed during a LD. The BAT is an organ with high oxidative activity that uses fatty acids as fuel [47–50]. Therefore, with the purpose of measuring this possible effect, we analysed the expression of genes related to thermogenesis, lipid uptake and beta-oxidation. *Lpl*, a gene implicated in lipid uptake, was down-regulated in the OSSD group compared to that in the VHSD group. Accordingly, OSSD animals also presented lower levels of *Cpt1b*, which controls the incorporation of fatty acids into the mitochondria for entry into the β-oxidation pathway [51]. These results indicate that less substrate is assimilated into the adipocytes, and thus, less fat is burned [48]. The same effect was observed for *Pparα* gene expression, a nuclear receptor found in BAT that induces several other genes for thermogenesis and lipolysis [52]. These effects indicate a down-regulation of BAT catabolic activity, which could contribute to the fat accretion observed in the adipose tissue. According to our hypothesis, rats consuming OS receive signals that the days are lengthening, and thus, OS consumption could activate a combination of fat accumulation signals, preparing animals for a long photoperiod. Koojiman et al. [35] demonstrated that the inactivation of BAT in rats in an LD photoperiod was responsible for the increase in body fat mass and decrease in triglyceride uptake in BAT, which supports the changes that we have observed. Surprisingly, we observed a higher *Ucp1* expression level in the OSSD group than the control. These results were unexpected because BAT has been reported to uptake plasma triglycerides and to metabolize lipids as fuel for thermogenesis [48, 53, 54], so we would expect to have a similar gene expression profile as the other lipid uptake and β-oxidation genes. Thus, we assumed that *Ucp1* would be down-regulated by OS in a SD. However, Western blotting did not confirm the influence of OSSD on *Ucp1*, so even though *Ucp1* expression in the OSSD group was up regulated, the protein levels were non-correlative. Takahashi et al. [55] demonstrated in mice fed a high-fat diet that UCP1 can be highly regulated post-transcriptionally, and other authors have reported several miRNA with suppression capabilities for BAT translation and/or metabolism [56, 57], which could be the reason for our divergent results between *Ucp1* gene expression and protein content in the OSSD group.

In conclusion, in our study we obtained evidence that the consumption of navelina orange harvested in the spring from the southern hemisphere increases the fat content of rats held in a short photoperiod (which represents the fall season), increasing lipogenesis, the percentage of large adipocytes and down-regulating BAT lipid uptake and β-oxidation gene expression. We propose that these effects are produced by the specific quantity of each polyphenol and/or particular phenolic composition of the fruit, which according to our hypothesis and the xenohormesis theory, has a different molecular signature depending on the season of growth, creating a physiological response in the rats. In our case, the OS would signal the advance of a long photoperiod, which translates to an increase in fat accumulation via a decrease in BAT activity, allowing rats to adapt beforehand to the new setting. These findings provide a new vision of diet and its influence on the organism, adding food origin and season to the numerous factors that must be taken into account in health and dietary recommendations to prevent obesity and the metabolic syndrome. However, it must be taken into account that this is an exploratory study and more evidence is needed. Future studies should focus on other fruits, how the environment specifically affects the polyphenol content in fruits and how this directly modifies animal physiology.

## Electronic supplementary material

Below is the link to the electronic supplementary material.

Supplementary material 1 (DOCX 13 KB)
