# Supplementary material for: Consumption of out-of-season orange modulates fat accumulation, morphology and gene expression in the adipose tissue of Fischer 344 rats
Source: Eur J Nutr. 2019 Feb 20;59(2):621–31. doi: 10.1007/s00394-019-01930-9 (PMC7058598; doi:10.1007/s00394-019-01930-9)
Supplement: Supplementary file 1 — Supplementary material 1 (DOCX 13 KB) [file 394_2019_1930_MOESM1_ESM.docx]

| **Supplementary table 1**. Primers for the Q-PCR analysis. | | |
| --- | --- | --- |
|  | **Forward (5'...3')** | **Reverse (5'...3')** |
| *Hprt* | TCCCAGCGTCGTGATTAGTGA | CCTTCATGACATCTCGAGCAAG |
| *Actb* | GCAGGAGTACGATGAGTCCG | ACGCAGCTCAGTAACAGTCC |
| *Ppia* | CTTCGAGCTGTTTGCAGACAA | AAGTCACCACCCTGGCACATG |
| *Acacα* | GCGGCTCTGGAGGTATATGT | TCTGTTTAGCGTGGGGATGT |
| *Atgl* | GAAGACCCTGCCTGCTGATT | CACATAGCGCACCCCTTGAA |
| *Fasn* | TAAGCGGTCTGGAAAGCTGA | CACCAGTGTTTGTTCCTCGG |
| *Gpat* | GAATACAGCCTTGGCCGATG | GAGGCGTGCATGAATAGCAA |
| *Hsl* | AGTTCCCTCTTTACGGGTGG | GCTTGGGGTCAGAGGTTAGT |
| *Prdm16* | GTTCTGCGTGGATGCCAATC | TGGCGAGGTTTTGGTCATCA |
| *Cebpα* | TGTACTGTATGTCGCCAGCC | TGGTTTAGCATAGACGCGCA |
| *Mgl* | ATCATCCCCGAGTCAGGACA | TGACTCCCCTAGACCACGAG |
| *Ucp1* | GGTACCCACATCAGGCAACA | TCTGCTAGGCAGGCAGAAAC |
| *Lpl* | GGCCCAGCAACATTATCCAG | ACTCAAAGTTAGGCCCAGCT |
| *Had* | ATCGTGAACCGTCTCTTGGT | AGGACTGGGCTGAAATAAGG |
| *Cpt1b* | GCAAACTGGACCGAGAAGAG | CCTTGAAGAAGCGACCTTTG |
| *Pparα* | CGGCGTTGAAAACAAGGAGG | TTGGGTTCCATGATGTCGCA |
| *Fatp1* | CTACCACTCAGCAGGGAACA | GCGGCATATTTCACCGATGT |
| *Cd36* | CAGTGCAGAAACAGTGGTTGTCT | TGACATTTGCAGGTCCATCTATG |
| *Pparγ* | AGGGCGATCTTGACAGGAAA | CGAAACTGGCACCCTTGAAA |
